# Protection of Gueichih-Fuling-Wan on cerebral ischemia-induced brain injury in rodents is mediated by trans-cinnamaldehyde via inhibition of neuroinflammation and apoptosis

**DOI:** 10.37796/2211-8039.1449

**Published:** 2024-06-01

**Authors:** Yuh-Fung Chen, Kuo-Jen Wu, Chi-Chung Kuo, Huei-Yann Tsai

**Affiliations:** a Department of Pharmacology, China Medical University, Taichung 404328, Taiwan; b Department of Pharmacy, China Medical University Beigang Hospital, Yunlin County 651012, Taiwan; c Department of Pharmacy, China Medical University, Taichung 406040, Taiwan; d Department of Neurology, Taichung Tzu Chi Hospital, Taichung 427003, Taiwan; e School of Post-Baccalaureate Chinese Medicine, Tzu Chi University, Hualien 970374, Taiwan; f Department of Pharmacy, China Medical University Hospital, Taichung 404327, Taiwan

**Keywords:** Gueichih-Fuling-Wan (GFW), Trans-cinnamaldehyde (TCA), Cerebral, Ischemia, Neuroinflammation and apoptosis, Neuroprotection

## Abstract

**Background:**

Stroke is the leading cause of mortality and morbidity worldwide, and an effective therapeutic strategy for the prevention of patients with cerebral ischemia induced brain injury is lacking. Traditional Chinese medicine with neuroprotective activities might be beneficial and provide alternative therapeutic opportunities for cerebral ischemia.

**Purposes:**

This study aimed to evaluate the neuroprotection and possible mechanisms of Gueichih-Fuling-Wan (GFW), its’ constitutive herbs, and their active compounds on cerebral ischemia/reperfusion (I/R)-induced brain injury in rodents.

**Methods:**

Various doses of extracts (0.25, 0.5, and 1.0 g/kg) of GFW and five constituent herbs (*Cinnamomi Cortex*, CC; *Poria cocos*, PC; *Paeonia lactifloa*, PL; *Paeonia suffruticosa*, PS and *Prunus perisica*, PP) were orally administered. Different doses of active compounds (0.5, 1.0, and 2.0 mg/kg) of GFW such as cinnamaldehyde, cinnamic acid (from CC), paeoniflorin (from PL), and paeonol (from PS) were intraperitoneally administered. Their effects on cerebral ischemia/ reperfusion (I/R)induced brain injury in rodents were evaluated.

**Results:**

GFW, its’ constituent herbs, and the active compounds reduced the infarct area dose-dependently (***P < 0.001). Cinnamaldehyde showed the most significant reduction (***P < 0.001). Therefore, trans-cinnamaldehyde (TCA) was further used to evaluate the neuroprotective mechanism of the I/R-induced brain injury. TCA (10, 20, 30 mg/ kg, p.o.) showed an inhibitory effect of I/R-induced brain damage in mice in a dose-dependent manner. Besides, GFW and TCA dose-dependently reduced the COX-2 protein expression level, and TCA reduced the TUNEL (+) apoptosis. TCA dose-dependently increased the pro-survival NR2A and Bcl-2 protein expression level and decreased the pro-apoptotic NR2B and cytochrome *c*, caspase 9, and caspase 3 expression (***P < 0.001).

**Conclusion:**

The above data revealed that GFW, its’ constituent herbs, and active compounds protected against I/R-induced brain injury in rodents. TCA from CC might participate in GFW protecting against cerebral ischemia-induced brain injury by inhibiting neuroinflammation and apoptosis.

## 1. Introduction

Stroke is the second-leading cause of death and the third-leading cause of disability and death in the world. There are currently 16 million stroke patients in the world, and the number of stroke patients is growing at a rate of 6 million every year [1–3]. The mortality rate within 30 days after stroke is approximately 24.6%, and 61% of stroke patients become disabled within 12 months after stroke. All these lead to severe socioeconomical burdens [4–6]. Ischemic stroke, which is approximately 85% of all causes of stroke, results in insufficient oxygen and glucose delivery to support cellular hemostasis [7], which causes injury to neurons, glia within the core of ischemic territory, excitotoxic and necrotic cell death that leads to brain injury. Cell death following stroke is associated with excitotoxicity, inflammation, oxidative stress, and apoptosis [7]. Due to the complex interplay events during ischemia, targeting these mechanisms is essential to provide therapeutic opportunities. Effective neuronal protection therapy may be an alternative strategy for cerebral ischemia.

Traditional Chinese medicine has been described as a treatment for various ailments associated with stroke in ancient medicine systems. Guizchih-Fuling-Wan (GFW), a famous traditional formula, comprises five Chinese medicines, including *Cinnamomum cassia* Blume (CC), *Poria cocos* (Schw.) Wolf (PC), *Paeonia lactifloa* Pall. (PL), *Paeonia suffruticosa* Andr. (PS), *Prunus perisica* (L.) Batsch (PP) [8] and major compounds including cinnamaldehyde, cinnamic acid, paeoniflorin and paeonol [9]. GFW has mainly been used to treat gynecological diseases for thousands of years and is used for various gynecological conditions in modern clinical practice, such as primary dysmenorrhea [10] and uterine fibroids [11], cervical cancer [12], and ovarian cancer [13]. No paper reports the protective effects of TCM from the formula, to its component herbs and the major compounds in one study. The present study aimed to determine the protective effect of GFW, its component herbs and major compounds on cerebral I/R induced brain injury and its possible action mechanisms. Among the major compounds, the most active compound was used for the study of possible action mechanism.

## 2. Materials and methods

### 2.1. Extraction of GFW and its component herbs

GFW comprises five Chinese herbs, including CC, PC, PL, PS, and PP, at a ratio of 1:1:1:1:1. Each herb medicine was 200 g and extracted twice with 2 L boiling water for 2 h. The extracts were filtered and freeze-dried for use. The yield of extracts was 11.54% and GFW was freshly prepared in distilled water before experiment.

### 2.2. Chemicals and reagents

The chemicals were purchased from the following companies. Anti-caspase-3 (1:2000; GTX73090) and anti-caspase-9 (1:1000; GTX50676) antibodies were from GeneTex Inc. (Irvine, CA, USA). Anti-COX-2 (1:1000; ab179800), anti-NR2A (1:1000; ab169873) and anti-NR2B (1:1000; 28373) antibodies were from Abcam Inc. (Abcam Inc., Cambridge, UK). Anti-Bcl-2 (1:1000; sc-7382), anti-cytochrome C (1:500; sc13156), and anti-β-actin (1:1000; sc-47778) antibodies were from Santa Cruz Biotechnology (Santa Cruz, CA, USA). 2, 3, 5-triphenyltetrazolium (TTC), paeoniflorin, paeonol, cinnamaldehyde and cinnamic acid were from Sigma–Aldrich (St. Louis, MO, USA). The BCA assay kit (Pierce Biotechnology Inc., Rockford, IL, USA). DMEM, penicillin/streptomycin, FBS, and glutamine were from Thermo-Fisher (Thermo Fisher Scientific Inc., Waltham, PA, USA). Zoletil® was purchased from Virbac Laboratories (Carros, France). In Situ Cell Death Detection Kit, Fluorescein, following the manufacturer’s instructions (Merck KGaA, Darmstadt, Germany). DAPI (4, 6-diamidino-2-phenylindole, Thermo Fisher Scientific Inc., Waltham, PA, USA).

### 2.3. Animals and cerebral ischemia/reperfusion model

Eight-week-old Sprague–Dawley (SD) rats were purchased from BioLASCO Co. Ltd. (Taipei, Taiwan) and C57BL/6JNarl mice were purchased from NAR Labs (Taipei, Taiwan). All animals were fed with standard chow, housed at a constant temperature and relative humidity (22 ± 1 °C and 55 ± 5%, respectively) with 12 h inverted light–dark cycle for one week before the experiments. Four to six animals were used to obtain constant data in each group. Surgery was performed under Zoletil® anesthesia. The animal surgery protocol of ischemia/reperfusion was as previously reported [14]. The Institutional Animal Care and Use Committee (IACUC) of China Medical University reviewed and approved this animal experimental protocol (CMU-IACUC96-77-N).

GFW (0.25, 0.5, and 1.0 g/kg) and each component herb (PL, CC, PP, PS, PC) (50, 100, 200 mg/kg) were orally administered 60 min before cerebral ischemia. Various dosages (0.5, 1.0, 2.0 mg/kg) of active compounds of GFW (cinnamaldehyde, cinnamic acid, paeonol, and paeoniflorin) were intraperitoneally injected 30 min before ischemia surgery.

### 2.4. Immunofluorescence staining of apoptotic cells

Mice were randomly assigned into control and trans-cinnamaldehyde (TCA)treated groups (n = 4). Group 1 mice were treated with saline, and groups 2, 3 and 4 were treated with TCA (10, 20, 30 mg/kg, p.o.). Cerebral ischemia/reperfusion (I/R) was induced as previously described [14]. Twenty-four h after I/R, the mice were anesthetized with Zoletil® (50 mg/kg, i.p.), intracardiac perfusion with 0.9% saline and 4% paraformaldehyde in 0.1 M PBS, then was decapitated as previously described [14].

The immunofluorescence staining of the brain slice was performed using the In Situ Cell Death Detection Kit, Fluorescence (Merck), following the manufacturer’s instruction manual. Images were captured with a fluorescence microscope. The percentage of TUNEL/DAPI-positive apoptotic cells in the cortex was estimated based on the average number of cells in a defined area.

### 2.5. Western blot analysis

Animals were sacrificed by intraperitoneal injection of Zoletil® (50 mg/kg), and the brain tissues were removed quickly and placed on ice. A homogenate (10%) was prepared in lysis buffer and centrifuged (12,000 rpm) for 30 min at 4 °C. A BCA protein assay kit with BSA standards determined sample protein concentrations. As previously reported, total protein (70 μg) was separated on 10% SDS-PAGE and transferred to PVDF membranes [14]. The membranes were then incubated with anti-COX-2, antiNR2A, anti-NR2B, anti-Bcl-2, anti-cytochrome C (cyto C), anti-caspase 3 (casp 3), anticaspase 9 (casp 9), and anti-β-actin. Immunodetection of bands was done using an enhanced chemiluminescence detection kit (Amersham Piscataway, NJ, USA).

### 2.6. Statistical analysis

All data were expressed as mean ± SE. Single variable comparisons used the Student’s *t*-test, and for variable comparisons used the one-way ANOVA followed by Dunnett’s test. When a *P* value < 0.05 was regarded as statistically significant.

## 3. Results

### 3.1. GFW alleviated cerebral I/R-induced brain injury in rats

**Fig. 1:**
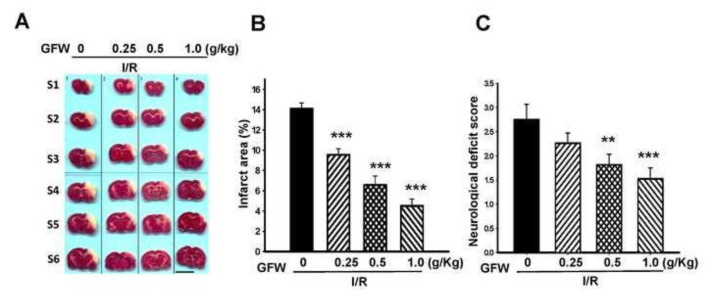
Effects of GFW on cerebral ischemia/reperfusion (I/R)-induced brain damage in rats. Cerebral infarct area was determined at 24 h after reperfusion. (A)TTC staining. Show the TTC stained sections of the rats in ischemia group, GFW 0.25 g/kg, 0.5 g/kg, and 1.0 g/kg group, respectively. The pale area represents infarct tissue and the red area normal tissue. (B) Infarct volume by TTC and (C) Neurological deficit score. Each bar represents mean ± S.E. In comparison with I/R group, after treatment of GFW (0.25–1.0 g/kg) (oral administration 1 h before ischemia), the infarct volume and neurological deficit were significantly decreased. **P < 0.01 and ***P < 0.001 compared to ischemia group. Scale bar = 1 cm(n = six per group).

Firstly, water extract of GFW was used to evaluate the protective effect on cerebral I/R–induced brain injury. Oral administration of GFW (0.25, 0.5, 1.0 g/ kg) 1 h before cerebral ischemia showed significant alleviation in the infarct area in a dose-dependent manner (***P < 0.001 compared with the I/R group) (Fig. 1A and B). The reduction rate was 31.49%, 52.48%, and 67.31%, respectively. GFW also effectively ameliorated neurological deficit scores (**P < 0.01 and ***P < 0.001, compared with the I/R group), as indicated in Fig. 1C. The reduction rate was 33.97% and 44.49%, respectively.

### 3.2. Component herbs of GFW ameliorated cerebral I/R-induced brain injury in rats

Secondly, water extracts of five component herbs of GFW were used to evaluate the protective effect on cerebral I/R-induced brain injury. Different doses (50, 100, 200 mg/kg, p.o.) of five component herbs, including PL, CC, PP, PS, and PC, showed amelioration on cerebral I/R induced infarct area in a dose-dependent manner as indicated in Fig. 3. The reduction rate of various doses of PL was 33.04%, 47.43%, and 67.89%, respectively. The reduction rate of CC was 47.25%, 70.35%, and 75.77%, respectively. The reduction rate of PP was 43.97%, 63.3%, and 80.62%, respectively; the reduction rate of PS was 22.39%, 42.49%, and 63.46%, respectively; the reduction rate of PC was 56.12%, 46.83%, and 59.29%, respectively. Among the five component herbs of GFW, CC and PP were the two herbs that showed more significant amelioration (***P < 0.001) in the infarct area; however, CC was the most effective one.

### 3.3. Active compounds of GFW improved cerebral I/R-induced brain injury in rats

Thirdly, four major compounds of GFW were used to evaluate the protective effect on cerebral I/ R-induced brain injury. Various doses (0.5, 1.0, and 2.0 mg/kg, i.p.) of cinnamaldehyde, cinnamic acid, paeonol, and paeoniflorin were furtherly investigated for their effects on I/R-induced infarct area. As indicated in Fig. 4 cinnamaldehyde showed the most significant inhibition on infarct area, and the reduction rate was 48.46%, 66.97%, and 82.6%, respectively, compared with the I/R group. Cinnamic acid inhibited infarct area in 1.0 mg/kg and 2.0 mg/kg, ***P,0.001 compared with the I/R group). Paeonol dose-dependently alleviated infarct area, *P < 0.05 and ***P < 0.001 compared with the I/R group. Paeoniflorin inhibited the infarct area by reducing 17.19%, 25.88%, and 67.34% (*P < 0.05, *P < 0.05, and ***P < 0.001, respectively) compared with the I/R group.

### 3.4. GFW ameliorated COX-2 protein expression in I/R-induced brain injury in rats

**Fig. 2:**
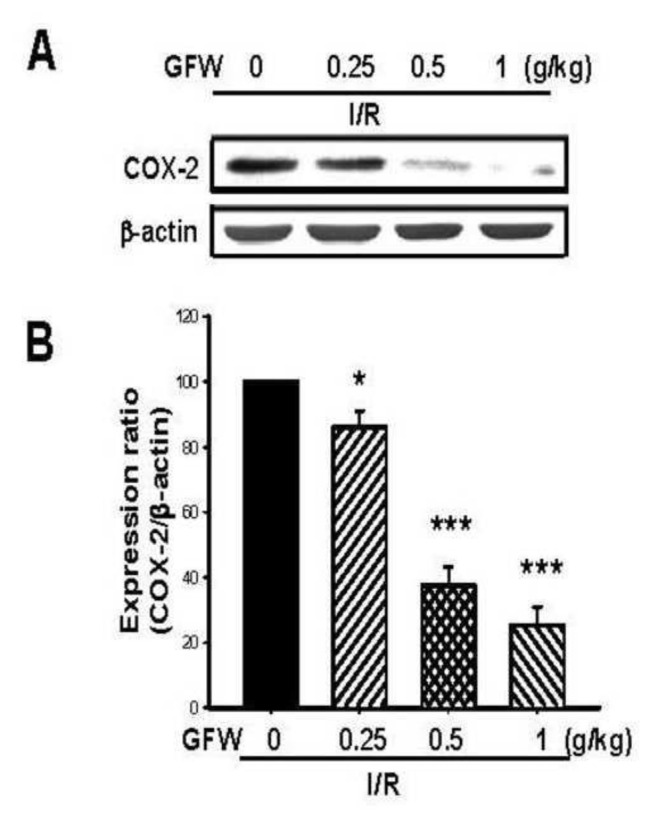
Effects of GFW on COX-2 protein expression level in I/R-induced brain tissue in rats. (A) COX-2 protein expression and (B) Protein expression ratio of COX-2/β actin. Bars represent mean ± S.E. from six independent experiments. *P < 0.05 and ***P < 0.001 compared to I/R group.

**Fig. 3:**
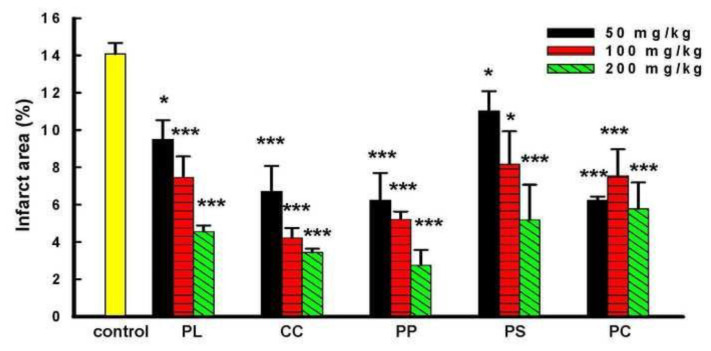
Effects of component herbs of GFW on I/R-induced brain damage in rats. Cerebral infract area was determined at 24 h after reperfusion. The TTC stained sections of the rats in ischemia control group, Paeoniala lactiflora (PL) group, Cinnamomum cassia (CC) group, Prunus persica (PP) group, Paeonia suffruticosa group, and Poria cocos (PC) group, respectively. The pale area represents infarct tissue and the red area normal tissue. Infarct volume by TTC staining (n = 6). Bars represent mean ± S.E. from six independent experiments. After treatment of PL, CC, PP, PS, and PC (50 ~200 mg/kg, p.o. 1 h before ischemia), the infarct volume significantly decreased. *P < 0.05 vs I/R group. ***P < 0.001 vs ischemia group.

**Fig. 4:**
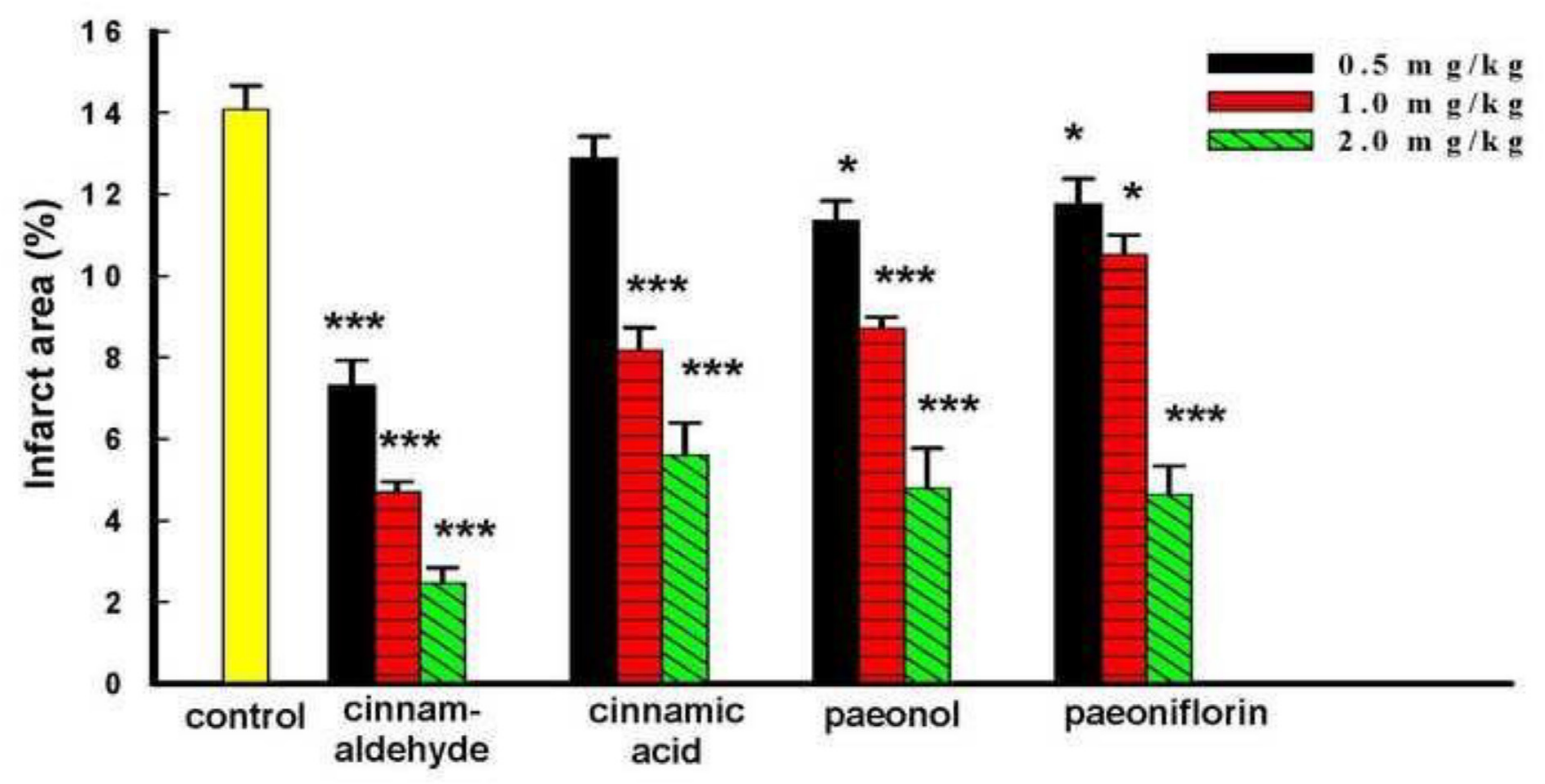
Effects of active compounds from component herbs of GFW on I/R-induced brain damage in rats. Cerebral infract area was determined at 24 h after reperfusion. The TTC stained sections of the rats in each group of ischemia control, cinnamaldehyde, cinnamic acid, paeonol, and paeoniflorin. Various dosage (0.5–2.0 mg/kg) of active compound intraperitoneal injection 30 min before ischemia, respectively. Infarct volume by TTC staining. Bars represent mean ± S.E. from six independent experiments. The infarct volume significantly decreased. *P < 0.05 vs ischemia group. **P < 0.01 vs ischemia group. ***P < 0.001 compared with I/R group.

In order to evaluate the amelioration of GFW on I/ R-induced inflammation in rat brains, COX-2 protein expression was assessed. Fig. 2A and B revealed that GFW (0.25, 0.5, 1 g/kg, p.o.) reduced COX-2 protein expression dose-dependently in the ischemic rat brain. The reduction rate was 13.93%, 62.60%, and 74.8%, respectively.

### 3.5. TCA alleviated infarct area in I/R-induced brain in mice

**Fig. 5:**
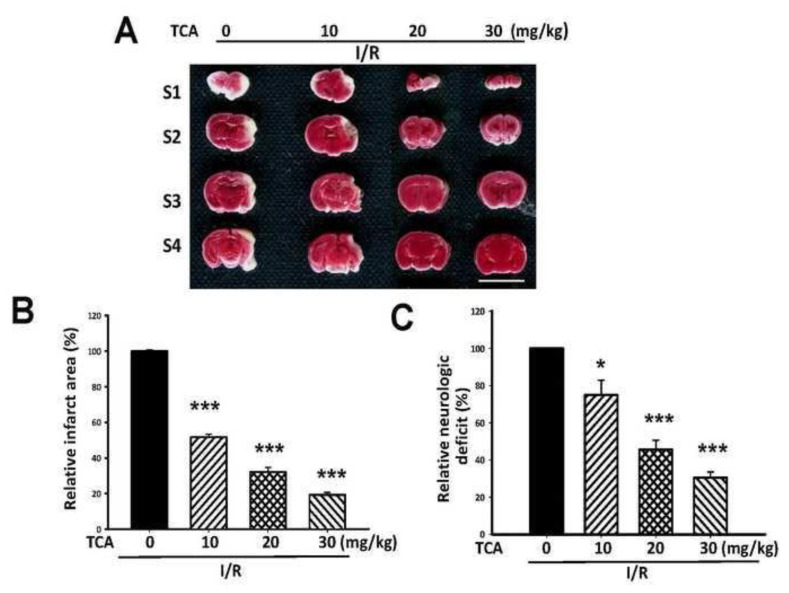
Effects of trans-cinnamaldehyde (TCA) on I/R-induced brain damage in mice. Cerebral infarct area was determined at 24 h after reperfusion. Various dosage of TCA (10, 20, 30 mg/kg) was orally administered. (A)TTC staining. Show the TTC stained sections of the mice in I/R group, TCA 10 mg/kg, 20 mg/kg, and 30 mg/kg group, respectively. The pale area represents infarct tissue and the red area normal tissue. (B) Infarct volume by TTC and (C) Neurological deficit score. Each bar represents mean ± S.E. In comparison with ischemia group, after treatment of TCA (10~30 mg/kg), the infarct volume and neurological deficit were significantly decreased. **P < 0.01 and ***P < 0.001 compared to ischemia group. Scale bar = 1 cm (n = four per group).

Since cinnamaldehyde showed the most significant amelioration on infarct area in I/R-induced brain injury in rats, the active form of cinnamaldehyde, transcinnamaldehyde (TCA), was further used to evaluate its effect on I/R-induced brain damage in mice. As indicated in Fig. 5A, TCA (10, 20, and 30 mg/kg, p.o.) showed a significant reduction of infarct area in I/R-induced mice brain damage. TCA significantly reduced infarct area and neurological deficit score (Fig. 5B and C) in a dose-dependent manner, and the relative infarct area reduction rate was 48.35%, 67.87%, and 80.67%, respectively. The relative neurologic deficit reduction rate was 25.15%, 54.39%, and 69.45%, respectively.

### 3.6. TCA ameliorated COX-2 protein expression in I/R-induced brain injury in mice

**Fig. 6:**
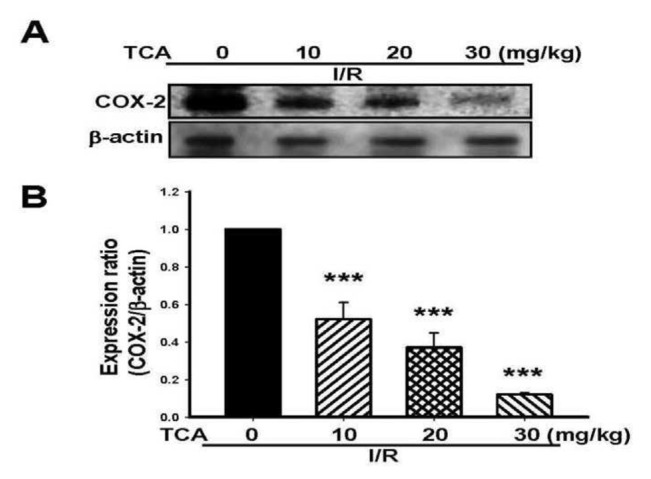
Effects of TCA on COX-2 protein expression level in I/R-induced brain tissue in mice. (A) COX-2 protein expression and (B) Protein expression ratio of COX-2/β-actin. Bars represent mean ± S.E. from six independent experiments. ***P < 0.001 compared to ischemia group.

In order to evaluate the amelioration of TCA on I/ R-induced inflammation in mice brains, COX-2 protein expression was assessed. TCA dose-dependently ameliorated the COX-2 expression in I/R-induced brain damage (***P < 0.001, compared with the I/R group), as indicated in Fig. 6A and B. The reduction rate of COX-2 protein expression was 48%, 63%, and 88%, respectively.

### 3.7. TCA alleviated apoptotic cells in I/R-induced mice brain injury

**Fig. 7:**
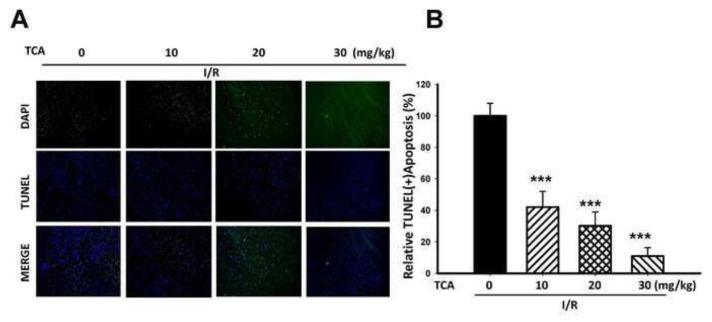
Effects of TCA on I/R-induced brain damage in mice. (A) The presence of neuronal apoptosis within the cortex was determined using immunofluorescence staining of TUNEL/DAPI. Representative images of the nucleus was stained with DAPI (blue), and the cells were stained with TUNEL (green). (B) Relative percentage of apoptotic cells in the brain of I/R mice. TCA significantly reduced apoptotic cells compared to I/R group (***P < 0.001 compared to ischemia group).

After I/R, complex mechanisms are involved in brain injuries, including neuron apoptosis. The number of apoptotic neurons in the ischemic boundary zone was assayed by the DAPI/TUNEL method. The presence of neuronal apoptosis within the cortex areas was determined using a TUNEL assay and DAPI staining. TCA treatment (10, 20, and 30 mg/kg, p.o.) significantly alleviated TUNEL (+) apoptotic cells compared with the I/R group (Fig. 7A). The reduction rate of TUNEL (+) apoptosis was 58%, 69.8%, and 89.1%, respectively (***P < 0.001) in a dose-dependent manner as indicated in Fig. 7B.

### 3.8. TCA reduced apoptotic pathway related protein expression in I/R-induced brain injury in mice

**Fig. 8:**
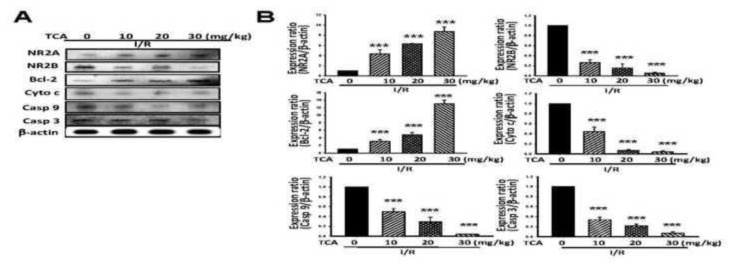
Effects of TCA on cell death pathway-relative protein expression in I/R-induced brain damage in mice. (A) Cell death pathway-relative protein expression, (B) NR2A, NR2B, Bcl-2, cytochrome (cyto) c, caspase (casp) 9, and casp 3. Bars represent mean ± S.E. from at least four independent experiments. ***P < 0.001 compared to I/R group.

The ischemia-induced mitochondrial dysfunction and opening of mitochondrial permeability transition pore (MTPT) lead to the release of cytochrome *c* and other proapoptotic factors, leading to activate caspase 9 and caspase3. As indicated in Fig. 8A, TCA treatment significantly reduced the neuronal apoptotic proteins such as NR2B, cyto C, casp 9, and casp 3. As indicated in Fig. 8B, the reduction rate was 74%, 85%, and 95% in NR2B, respectively, and reduced cyto C by 55.63%, 93.28%, and 96.23%, respectively. The reduction rate of casp 9 was 50.26%, 70.74%, and 96.23%, respectively, and the reduction rate of casp 3 was 66%, 78%, and 93.07%, respectively (see Fig. 8).

### 3.9. TCA enhanced neuronal survival-related protein expression in I/R-induced brain injury in mice

As indicated in Fig. 8, TCA treatment (10, 20, and 30 mg/kg, p.o.) significantly increased the neuronal survival protein expression level (NR2A and Bcl-2). The increase rate was 334%, 531%, and 741% in NR2A, respectively. Furthermore, the increase rate in Bcl-2 was 211%, 376%, and 1200%, respectively.

## 4. Discussion

Stroke is a crucial cause of mortality and morbidity worldwide. Stroke can be subdivided into two categories: ischemic and hemorrhagic stroke. Ischemic stroke is the primary type and makes up approximately 85% of all cases, which have been the target of most drug trials [15]. A thrombosis restricts blood flow to the brain and causes insufficient oxygen and glucose supply to support cellular homeostasis [16]. These processes share overlapping biochemical abnormalities causing injury to neurons, glia, and endothelial cells. Within the core of the ischemic territory, the blood flow is severely restricted, and excitotoxic and necrotic cell death occurs within minutes [17]. Thus, it elicits multiple processes, such as free radical production, excitotoxicity, ionic imbalance, oxidative stress, inflammation, and apoptosis, that lead to brain injury [16,18]. Using tissue plasminogen activator (tPA) to treat stroke patients is the current therapeutic strategy. However, tPA treatment confronts ill side effects, such as tPA is limited to stroke patients within 4.5 h of stroke incidence and increases the danger of cerebral bleeding and brain damage, thus limiting their usage in clinical settings [19]. Owing to the difficulties associated with tPA, it is necessary to consider other safe and efficacious strategies for stroke. The availability of novel adjuvant neuroprotective tools may lead to a paradigm shift in stroke therapy [19]. Recent evidence has indicated that monoclonal antibodies targeting an endogenous molecule or signaling cascade would reduce the severity of the damaged brain tissue; however, the clinical evidence for stroke therapy is minimal [20]. Although monoclonal antibody therapy has proven effective and safe in treating various neurological disorders in animal studies, the complete antibody cannot cross the blood–brain barrier (BBB), and immunogenicity is a significant limitation [20]. Many studies are currently devoted to ascertaining safer and more effective therapies for ischemic stroke to meet clinical needs, and stem cell based ischemic stroke therapy is a current trend [21]. However, the medical expense of either monoclonal antibody or stem-cell therapies is a heavy burden for patients. Therefore, seeking a safe and affordable therapeutic strategy is necessary.

Traditional Chinese medicine has been described as a treatment for various ailments associated with stroke in ancient medicine systems. It is a complementary and alternative therapy to make up for the deficiencies of Western medicine. Evidence reveals that TCM exerts anti-inflammatory effects in experimental cerebral ischemia by inhibiting inflammatory mediators, leukocyte infiltration, and BBB disruption, which indicates TCM may play a role in ischemia [22].

Previous reports indicated that GFW inhibited atherosclerosis [23,24], protected against chemical damage to brain neurons [25], and improved diabetes and related complications [23,25,26]. Our group reported that GFW is neuroprotective on cerebral ischemia/reperfusion injury in streptozotocin (STZ)-induced hyperglycemic rats [9,27].

STZ-induced hyperglycemia stimulates apoptotic pathways, leading to neuronal cell death [28,29]. Apoptosis occurs in several neurodegenerative diseases, such as ischemic stroke, Alzheimer’s disease, and Parkinson’s disease [30]. Caspase enzymes and Bcl-2 family members are the critical elements in apoptosis; caspase 3 plays a pivotal role in apoptosis [31] and Bcl-2 is the antiapoptotic protein [32]. GFW treatment reduced caspase-3 protein levels and increased levels of the antiapoptotic protein Bcl2, indicative of neuroprotection. The protective effects of GFW on neuronal apoptosis and cognition deficits caused by STZ-induced hyperglycemia may be partly due to inhibition of the cellular apoptosis pathway [9]. In the process of brain ischemia induced neuronal cell death, excessive generation of nitric oxide (NO) free radicals is implicated in the neurotoxicity. GFW protected against nitric oxide-mediated neuronal death in cultured cerebellar granule cells, and it was derived from Cinnamomi Cortex (CC), Paeoniae Radix (PL), and Moutan Cortex (PS) reported by Shimada et al. [26] that supported our data. Chen et al. previously reported that GFW significantly reduced LPS-induced NO production and attenuated iNOS and COX-2 expression in LPS treated BV-2 cells [27]. GFW protects against cerebral I/R injury in hyperglycemic rats; due to inhibition of cellular apoptosis and neuroinflammation [27].

In this study, GFW showed significant amelioration in the infarct area and neurological deficit score; the component herbs also showed a similar inhibition on I/R induced brain damage. It indicated that GFW and its constituent medicinal plants showed neuroprotection on neuronal death. Chen et al. reported PL and paeoniflorin significantly attenuated cerebral infarction in I/R injury rats and the severity of carotidligation-induced intimal hyperplasia in mice, which was related to the modulation of the Ras/MEK/ERK signaling pathway [33]. Hsieh et al. reported that paeonol reduced cerebral infarct and neuro-deficit in I/R rats, suppressed and scavenged superoxide anion, and inhibited microglia activation and IL-1β in I/R rats [34]. GFW and five constituent herbs dose-dependently inhibited NO production in LPS-treated BV2 microglial cells; CC showed the most significant inhibition (authors’ unpublished data). Chen et al. reported that TCA inhibited LPS-induced NO production in BV-2 microglial cells and reduced iNOS and COX-2 expression [35].

Moreover, TCA suppressed LPS-induced nuclear translocation of NF- κB p65 and p50 and increased cytosolic IκBα TCA inhibited NO production and inflammation by down-regulating iNOS, COX-2, and TNF-α gene expression and suppressing the NF κB and p53 pathways [35]. COX-2 plays a vital role in cerebral and neuroinflammation, which upregulates COX-2 expression in neurons, glia, and inflammatory cells invading the ischemic brain [36]. Thus, COX-2 is an attractive target for stroke therapy [36]. In cerebral ischemia, COX-2 contributes to glutamate excitotoxicity [37,38], a vital factor in the Ca^2+^ dysregulation initiating the ischemic cascade [39].

TCA protects against I/R-induced brain damage by reducing the TUNEL (+) apoptosis and COX-2 protein expression level in I/R-induced damaged brain. GFW and TCA showed a significant reduction of COX-2 protein expression level, revealing that inhibition of COX-2 attenuates I/R-induced brain damage attributed to the neuroprotection of GFW and TCA. The above data implied that TCA mediated the neuroprotection of GFW.

Nuclear factor E2-related factor (Nrf2) is a vital endogenous antioxidant protein that combines with Keap 1 to maintain a dormant state under physiological conditions. When I/R occurs, Nrf2 dissociates from Keap1 and activates the expression of down-stream antioxidant protein to exert a protective effect. After cerebral I/R, oxidative stress and inflammation activate macrophages to secret more TNF-α. The rising TNFα combines with TNF-R1 receptors to recruit and activate initiator caspase 8. Then, the activated caspase 8 activates caspase 3 and incurs an exogenous apoptosis reaction [40–42]. It is reported that Nrf2 activation can inhibit the activation of the NF-κB pathway, decreasing the secretion of TNF-α and the trimerization of TNF-R1, restraining the activation of caspase 8 and caspase 3 [43,44]. Current studies showed that activation of the Nrf2/ HO-1 pathway enhances Bcl-2 to combine with Bax, prevents Bax from forming oligomer and creating pores on the mitochondrial outer membrane, reduces the release of AIF and cytochrome *c* (cyto c), inhibits the initiator caspase 9 (casp 9) activation, and further blocked executioner caspase 3 (casp 3) and therefore attenuated cell apoptosis [43,45,46]. Cyto c, casp 9 and casp 3 are involved in the mitochondria mediated procaspase-activation pathway [47]. Moreover, caspase enzymes and the Bcl2 family are the key elements in apoptosis and anti-apoptosis. In this study, the protective effects of TCA are related to the attenuation of cyto c, caspase 9, and casp 3 expression and enhanced the expression Bcl-2. Evidence suggests that Nrf2 activators play a vital role in treating cerebral I/R [48,49] and whether a correlation between TCA and Nrf2 drives our great attention. In order to clarify this correlation, more research will be studied in the future.

After cerebral ischemia, excessive activation of NMDA (*N*-methyl-d-aspartic acid) receptors is a crucial cause of ischemic injury [50,51]. However, the roles of NMDA receptors in cerebral ischemia are complex and may have different functional outputs, including both pro-death and pro-survival ionotropic signaling [51]. Synaptic activation of the NR2A subunit-containing NMDAR, leading to an activation of the pro-survival signaling proteins Akt, ERK, and CREB, and stimulation of NR2B subunit-containing NMDAR in the extrasynaptic sites triggers excitotoxic neuronal death via PTEN, cdk5, and DAPK1 [50,52,53]. TCA markedly increases the expression level of NR2A and decreases NR2B expression level, revealing its role in neuroprotection. Over-excitation of NMDA receptors results in mitochondrial dysfunction and leads to brain damage.

TCA significantly reduced the protein expression level of cyto c, casp 9, and casp 3 and markedly increased the Bcl-2 protein expression level, revealing that the apoptosis pathway modulation mediated neuroprotection of TCA. This finding is also supported by a previous report that indicates the inhibition of neuroinflammation of TCA is through attenuation of iNOS, COX-2 expression, and NFκ-B signaling pathway [35].

**Fig. 9:**
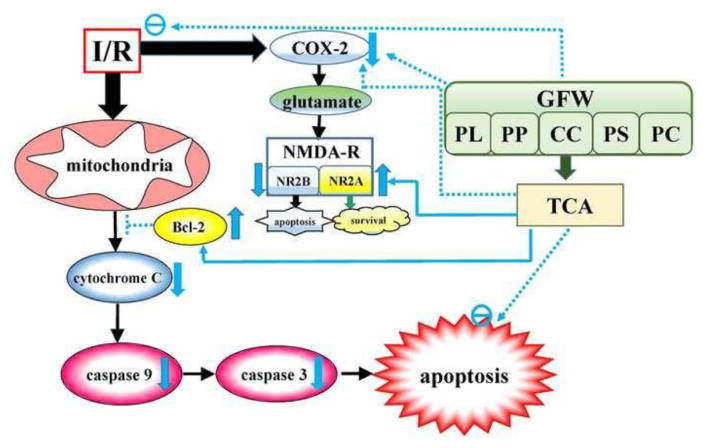
Proposed action mechanisms of GFW and TCA protect against I/R-induced brain injury. GFW and TCA protect against I-R-induced brain damage via the inhibition of neuroinflammation and apoptosis. Abbreviations-**GFW**: Gueichih-Fuling-Wan; **PL**: Paeonia lactifloa Pall; **PP**: Prunus perisica (L.) Batsch; **CC**: Cinnamomum cassia Blume; **PS**: Paeonia suffruticosa Andr; **PC**: Poria cocos (Schw.) Wolf; **TCA**: transcinnamaldehyde; **I/R**: cerebral ischemia/reperfusion; **NMDA-R**: N-methyl-d-aspartate receptor; **NR2A**: NMDA receptor 2A subunit; **NR2B:** NMDA receptor 2B subunit; **cyto c:** cytochrome c; **casp 9:** caspase 9; casp 3: caspase 3; **COX-2:** cyclooxygenase-2. Dashed line represents inhibitory effect; straight line represents increasing effect. Upward green arrow represents enhancing, whereas downward green arrow represents inhibitory effect. Green Circle represent inhibition.

In conclusion, this study demonstrated that GFW is neuroprotective against I/R induced brain damage, and its constitutive herbs contribute to this neuroprotection. Moreover, TCA from CC showed the most significant inhibitory effect on protecting against I/R-induced brain damage via the inhibition of neuroinflammation and apoptosis. The proposed action mechanism of GFW and TCA protect against I/R-induced brain injury as shown in Fig. 9. Adequate neuronal protection of GFW and TCA may have therapeutic potential in patients with cerebral ischemia and be an alternative strategy for cerebral ischemia-induced brain damage.
